# Global cross-sectional student survey on AI in medical, dental, and veterinary education and practice at 192 faculties

**DOI:** 10.1186/s12909-024-06035-4

**Published:** 2024-09-28

**Authors:** Felix Busch, Lena Hoffmann, Daniel Truhn, Esteban Ortiz-Prado, Marcus R. Makowski, Keno K. Bressem, Lisa C. Adams, COMFORT Consortium, Nitamar Abdala, Álvaro Aceña Navarro, Hugo J. W. L Aerts, Catarina Águas, Martina Aineseder, Muaed Alomar, Salita Angkurawaranon, Zachary G. Angus, Eirini Asouchidou, Sameer Bakhshi, Panagiotis D. Bamidis, Paula N. V. P. Barbosa, Nuru Y. Bayramov, Antonios Billis, Almir G. V. Bitencourt, Antonio JBollas Becerra, Fabrice Busomoke, Andreia Capela, Riccardo Cau, Warren Clements, Alexandru Corlateanu, Renato Cuocolo, Nguyễn N. Cương, Zenewton Gama, Paulo J. de Medeiros, Guillermo de Velasco, Vijay B. Desai, Ajaya K. Dhakal, Virginia Dignum, Izabela Domitrz, Carlos Ferrarotti, Katarzyna Fułek, Shuvadeep Ganguly, Ignacio García-Juárez, Cvetanka Gjerakaroska Savevska, Marija Gjerakaroska Radovikj, Natalia Gorelik, Valérie Gorelik, Luis Gorospe, Ian Griffin, Andrzej Grzybowski, Alessa Hering, Michihiro Hide, Bruno Hochhegger, Jochen G. Hofstaetter, Mehriban R. Huseynova, Oana-Simina Iaconi, Pedro Iturralde Torres, Nevena G. Ivanova, Juan S. Izquierdo-Condoy, Aidan B. Jackson, Ashish K. Jha, Nisha Jha, Lili Jiang, Rawen Kader, Padma Kaul, Gürsan Kaya, Katarzyna Kępczyńska, Israel K. Kolawole, George Kolostoumpis, Abraham Koshy, Nicholas A. Kruger, Alexander Loeser, Marko Lucijanic, Stefani Maihoub, Sonyia McFadden, Maria CMendez Avila, Matúš Mihalčin, Masahiro Miyake, Roberto Mogami, András Molnár, Wipawee Morakote, Issa Ngabonziza, Trung Q. Ngo, Thanh T. Nguyen, Marc Nortje, Subish Palaian, Rui PPereira de Almeida, Barbara Perić, Gašper Pilko, Monserrat LPuntunet Bates, Mitayani Purwoko, Clare Rainey, João C. Ribeiro, Gaston A. Rodriguez-Granillo, Nicolás Rozo Agudelo, Luca Saba, Shine Sadasivan, Keina Sado, Julia M. Saidman, Pedro J. Saturno-Hernandez, Gilbert M. Schwarz, Sergio M. Solis-Barquero, Javier Soto Pérez-Olivares, Petros Sountoulides, Arnaldo Stanzione, Nikoleta G. Tabakova, Konagi Takeda, Satoru Tanioka, Hans O. Thulesius, Liz N. Toapanta-Yanchapaxi, Minh H. Truong, Murat Tuncel, Elon H. C. van Dijk, Peter van Wijngaarden, Lina Xu, Tomasz Zatoński, Longjiang Zhang

**Affiliations:** 1 Department of Neuroradiology, Charité – Universitätsmedizin Berlin, Corporate Member of Freie Universität Berlin and Humboldt Universität Zu Berlin, Luisenstraße 7, 10117 Berlin, Germany; 2 Department of Diagnostic and Interventional Radiology, University Hospital Aachen, Aachen, Germany; 3 One Health Research Group, Universidad de Las Américas, Quito, Ecuador; 4 School of Medicine and Health, Department of Diagnostic and Interventional Radiology, Klinikum rechts der Isar, TUM University Hospital, Technical University of Munich, Munich, Germany; 5 School of Medicine and Health, Institute for Cardiovascular Radiology and Nuclear Medicine, German Heart Center Munich, TUM University Hospital, Technical University of Munich, Munich, Germany

**Keywords:** Artificial intelligence, Students, Medical, Education, Medical, Cross-sectional studies, Curriculum, Surveys and questionnaires

## Abstract

**Background:**

The successful integration of artificial intelligence (AI) in healthcare depends on the global perspectives of all stakeholders. This study aims to answer the research question: What are the attitudes of medical, dental, and veterinary students towards AI in education and practice, and what are the regional differences in these perceptions?

**Methods:**

An anonymous online survey was developed based on a literature review and expert panel discussions. The survey assessed students' AI knowledge, attitudes towards AI in healthcare, current state of AI education, and preferences for AI teaching. It consisted of 16 multiple-choice items, eight demographic queries, and one free-field comment section. Medical, dental, and veterinary students from various countries were invited to participate via faculty newsletters and courses. The survey measured technological literacy, AI knowledge, current state of AI education, preferences for AI teaching, and attitudes towards AI in healthcare using Likert scales. Data were analyzed using descriptive statistics, Mann–Whitney U-test, Kruskal–Wallis test, and Dunn-Bonferroni post hoc test.

**Results:**

The survey included 4313 medical, 205 dentistry, and 78 veterinary students from 192 faculties and 48 countries. Most participants were from Europe (51.1%), followed by North/South America (23.3%) and Asia (21.3%). Students reported positive attitudes towards AI in healthcare (median: 4, IQR: 3–4) and a desire for more AI teaching (median: 4, IQR: 4–5). However, they had limited AI knowledge (median: 2, IQR: 2–2), lack of AI courses (76.3%), and felt unprepared to use AI in their careers (median: 2, IQR: 1–3). Subgroup analyses revealed significant differences between the Global North and South (*r* = 0.025 to 0.185, all *P* < .001) and across continents (*r* = 0.301 to 0.531, all *P* < .001), with generally small effect sizes.

**Conclusions:**

This large-scale international survey highlights medical, dental, and veterinary students' positive perceptions of AI in healthcare, their strong desire for AI education, and the current lack of AI teaching in medical curricula worldwide. The study identifies a need for integrating AI education into medical curricula, considering regional differences in perceptions and educational needs.

**Trial registration:**

Not applicable (no clinical trial).

**Supplementary Information:**

The online version contains supplementary material available at 10.1186/s12909-024-06035-4.

## Background

The popularity of artificial intelligence (AI) in healthcare has exponentially risen in recent years, attracting the attention of professionals and students alike [1, 2]. The emergence of large language models like ChatGPT has further expanded AI’s potential in medicine, offering new possibilities for clinical applications and medical training [3, 4]. AI has demonstrated expert-level performance in various medical domains, including breast cancer screening, chest radiograph interpretation, and prediction of treatment outcomes [5–8].

The increasing prevalence of AI in healthcare necessitates its incorporation into medical education. AI offers numerous potential benefits for medical training, such as enhancing understanding of complex concepts, providing personalized learning experiences, and simulating clinical scenarios [9–12]. Moreover, familiarizing medical students with AI tools and technologies prepares them for the realities of their future professional lives [13, 14]. However, the integration of AI also raises significant ethical challenges, including concerns about patient autonomy, beneficence, non-maleficence, and justice [9, 15, 16].

Existing literature has primarily focused on the technical aspects of AI in medicine or its potential applications in specific medical specialties [17]. Other studies have explored healthcare professionals' perceptions of AI, but these have been limited by small sample sizes and lack of geographic diversity [17]. This gap in the literature precludes a comprehensive understanding of how future healthcare professionals across different regions perceive and prepare for AI integration in their fields.

This multicenter study addresses this gap by investigating the perspectives of medical, dental, and veterinary students on AI in their education and future practice across multiple countries. Specifically, we examine: 1) students' technological literacy and AI knowledge, 2) the current state of AI in their curricula, 3) their preferences for AI education, and 4) their attitudes towards AI's role in their fields. By exploring regional differences on a large, international scale, this study offers a unique comparative overview of students' perceptions worldwide.

## Methods

This multicenter cross-sectional study was conducted in accordance with the Strengthening the reporting of observational studies in epidemiology (STROBE) statement and received ethical approval from the Institutional Review Board at Charité – University Medicine Berlin (EA4/213/22), serving as the principal institution, in compliance with the Declaration of Helsinki and its later amendments [18, 19]. To ensure participant anonymity, the necessity for informed consent was waived.

### Instrument development and design

Following the Association for Medical Education in Europe (AMEE) guide, this study aimed to develop an anonymous online survey to assess: 1) the technological literacy and knowledge of informatics and AI, 2) the current state of AI in their respective curricula and preferences for AI education, and 3) the perspectives towards AI in the medical profession among international medicine, dentistry, and veterinary medicine students [20]. To inform instrument development, a literature review of existing publications on the attitudes of medical students towards AI in medicine was independently performed by four reviewers (FB, LH, KKB, LCA), leveraging MEDLINE, Scopus, and Google Scholar databases in December 2022. Studies were selected for review based on the following criteria: 1) the publications were original research articles, 2) the scope aligned with our research objectives and targeted medical students, 3) the survey was conducted in English language, 4) the items were publicly accessible, 5) the measurement of perspectives towards AI was not restricted to a particular medical subfield. Following these criteria, five articles comprising a total of 96 items were identified as relevant to the research scope [21–25]. After a consensus-based discussion, items that did not match our research objectives or overlapped in content were excluded, resulting in 23 remaining items. These items were subsequently tailored to fit the context of medical education and the medical profession.

A review cycle was undertaken with a focus group of medical AI researchers and students, as well as an expert panel including physicians, medical faculty members and educators, AI researchers and developers, and biomedical statisticians (FB, LH, DT, MRM, KKB, LCA, AB, RC, GDV, AH, LJ, AL, PS, LX). The finalized survey consisted of 16 multiple-choice items, eight demographic queries, and one free-field comment section. These items were further refined based on content-based domain samples, and responses were standardized using a four- or five-point Likert scale where applicable.

The preliminary assessment was conducted through cognitive interviews with ten medical students at Charité – University Medicine Berlin to evaluate the scale's comprehensiveness and overall length. The feedback resulted in two rewordings and one item removal, finalizing the survey with 15 multiple-choice items and eight demographic queries supported by one free-field comment section. The final questionnaire items and response options can be viewed in Table 1.

**Table 1:** Questionnaire items and response options

Items	Response options
University or College	[Free text field]
Country	[Free text field]
Gender:	Male / Female / Diverse / Prefer not to disclose
Age:	Years
Current course of study:	(Human) Medicine / Dentistry / Veterinary Medicine / Other
Current academic year:	Years
Total academic years:	Years
Which of these technical devices do you use at least once a week?	Smartphone / PC/laptop / Game console (e.g., PlayStation, Switch) / Tablet (e.g., iPad) / E-reader / Smartwatch / None
Have you already programmed code?	Yes / No
In which language(s) have you programmed code?	[Free text field]
How would you rate your general knowledge of artificial intelligence (AI)?	No knowledge (never heard of AI) / Little knowledge (e.g., documentary seen on TV) / Good knowledge (e.g., read several journal articles on AI) / Expert (e.g., involved in AI research/development)
What is your current general attitude toward your medical studies?	Extremely negative / Rather negative / Neutral / Rather positive / Extremely positive
What is your general attitude toward the application of artificial intelligence (AI) in medicine?	Extremely negative / Rather negative / Neutral / Rather positive / Extremely positive
As part of my studies, there are curricular events on artificial intelligence (AI) in medicine	No / Yes; 1–5 h in total / Yes; > 5–10 h in total / Yes; > 10–20 h in total / Yes; > 20 h in total
I would like to have more teaching on artificial intelligence (AI) in medicine as part of my studies	Completely disagree / Rather disagree / Neutral / Rather agree / Completely agree
What would you like to learn about artificial intelligence (AI) as part of your medical curriculum?	Theory and background (e.g., mathematical basics) / Practical skills (e.g., learning programming languages; solving medical problems with AI) / History and development / Legal and ethical aspects / Future perspectives of AI in medicine / No preference / Other / Nothing
What other things would you like to learn about artificial intelligence (AI) as part of your medical curriculum?	[Free text field]
What is your view on the influence of artificial intelligence (AI) on the profession of physicians? AI will affect the everyday life of physicians in a way that is…	Extremely negative / Rather negative / Neutral / Rather positive / Extremely positive
How would you rate artificial intelligence (AI) software being available to physicians as a second opinion on medical issues?	Extremely negative / Rather negative / Neutral / Rather positive / Extremely positive
Suppose an artificial intelligence (AI) makes a diagnosis. What would you prefer?	The decision path is presented clearly and comprehensibly (explainable AI), and the accuracy is high. / The decision path is not presented (black box), but the accuracy is higher
Suppose an artificial intelligence (AI) makes a diagnosis. What would you prefer?	The AI misses almost no diagnosis, but often gives a false alarm (i.e., a high sensitivity). / The AI almost never gives a false alarm, but sometimes misses a diagnosis (i.e., a high specificity). / The AI gives a false alarm about as often as it misses a diagnosis
How do you estimate the effect of artificial intelligence (AI) on the efficiency of healthcare processes in the next 10 years?	Great deterioration / Moderate deterioration / No effect / Moderate improvement / Great improvement
The use of artificial intelligence (AI) in medicine will increasingly lead to legal and ethical conflicts	Completely disagree / Rather disagree / Neutral / Rather agree / Completely agree
I think working with artificial intelligence (AI) as a physician is necessary to stay competitive	Completely disagree / Rather disagree / Neutral / Rather agree / Completely agree
With my current knowledge, I feel sufficiently prepared to work with artificial intelligence (AI) in my future profession as a physician	Completely disagree / Rather disagree / Neutral / Rather agree / Completely agree

Using REDCap (Research Electronic Data Capture) hosted at Charité – University Medicine Berlin, the English survey was subsequently disseminated through the medical student newsletter at Charité and deactivated after receiving responses from 50 medical students who served as the pilot study group and were not included in the final participant pool [26, 27]. After psychometric validation, participating sites distributed the REDCap online survey among medical, dental, and veterinary students at their faculty. Due to the large number of Spanish-speaking sites, a separate Spanish online version of the survey was employed using paired forward and backward translation with reconciliation by two bilingual medical professionals (LG, JSPO). Depending on their faculty location, participating sites distributed either the English or Spanish online survey via their faculty newsletters and courses using a QR code or the direct website link (non-probability convenience sampling). The survey was available for participation from April to October 2023.

Our data collection methodology was designed to mitigate several risks related to privacy, confidentiality, consent, transparency of recruitment, and minimization of harm, as highlighted before [28]. By using faculty newsletters and course distributions, we reduced the exposure of personal information on social media platforms, thereby maintaining a higher level of privacy. This method ensured that our participants' identities and responses were not publicly available or exposed to wider online networks. To further secure the data, the survey platform used was selected for its robust security features, including data encryption and secure storage. We explicitly informed participants about how their data would be used and protected, ensuring transparency and building trust.

Distributing the survey through official academic channels, such as faculty newsletters, implied a degree of formality and oversight, increasing the likelihood that participants were adequately informed of the study's intentions. By detailing the purpose of the study, the use of data and participants' rights on the first page of the survey, participants had to indicate their understanding and agreement by ticking an 'I agree' box before proceeding.

Using institutional channels for distribution provided a transparent and credible recruitment process that was likely to reach a relevant and engaged audience. We ensured that participants were aware that their participation was completely voluntary and that they could withdraw from the study at any time without penalty. We also provided contact details for participants to ask questions about the study, promoting openness and trust.

By avoiding the use of social media for recruitment, we eliminated the risk of participants' responses being exposed to their social networks, thereby protecting their privacy and reducing potential social risks. The content of the survey was carefully reviewed to ensure that no questions could cause distress or harm to participants. Participants were informed that they could skip any questions they felt uncomfortable answering, ensuring their well-being and autonomy throughout the survey process.

### Inclusion and exclusion criteria

Inclusion criteria consisted of students at least 18 years of age, actively enrolled in a (human) medicine, dentistry, or veterinary medicine degree program, who responded to the survey during its open period and were proficient in either English or Spanish, depending on their faculty location. Participants had to confirm their enrollment in a relevant program and input their age to verify they were above 18 years old. Only those meeting these criteria could proceed with the survey. Respondents who started the survey but did not answer any multiple-choice items were excluded from the analysis. Partial missing responses to survey items resulted in exclusion from each subanalysis.

### Statistical analysis

Statistical analyses were performed with SPSS Statistics 25 (version 28.0.1.0) and R (version 4.2.1), using the "tidyverse", "rnaturalearth", and "sf" packages [29–32]. The Kolmogorov–Smirnov test was used to test for normal distribution. Categorical and ordinal data were reported as frequencies with percentages. Medians and interquartile ranges (IQR) were reported for non-parametric continuous data. Variances were reported for items in Likert scale format. The response rate was derived from the overall student enrollment numbers at each faculty according to the faculty websites or the Times Higher Education World University Rankings 2024 due to the unavailability of official data on enrolled medical, dentistry, or veterinary students. In the pilot study group, item reliability was measured using Cronbach's α, with values above 0.7 interpreted as acceptable internal consistency. Explanatory factor analysis was used to examine the structure and subscales of the instrument, using an eigenvalue cutoff of 1 for item extraction. Items with factor loadings of 0.4 or higher were retained. Data suitability for structural evaluation was assessed using the Kaiser–Meyer–Olkin measure and Bartlett's test of sphericity. For geographical subgroup analysis, respondents were categorized based on their faculty location (Global North versus Global South) according to the United Nations' Finance Center for South-South Cooperation [33]. Additionally, participants were grouped into continents based on the United Nations geoscheme [34]. Due to the substantial number of European participants, students in North/West and South/East Europe were analyzed separately. Further subgroup analyses based on gender, age, academic year, technological literacy, self-reported AI knowledge, and previous curricular AI events can be found in the appendix (see Supplementary Tables 1–7). The Mann–Whitney U-test was employed for subgroup analyses of two independent non-parametric samples. For continental comparison, the Kruskal–Wallis one-way analysis of variance and Dunn-Bonferroni post hoc test were performed. To estimate effect size, we calculated *r*, with 0.5 indicating a large effect, 0.3 a medium effect, and 0.1 a small effect [35]. An asymptotic two-sided *P*-value below 0.05 was considered statistically significant.

## Results

### Pilot study

The median age of the pilot study group was 24 years (IQR: 21–26 years). 58% of participants identified as female (*n* = 29), 38% as male (*n* = 19), and 4% (*n* = 2) did not report their gender. The median current academic year was 2 (IQR: 2–4 years) out of 6 total academic years. Internal consistency for our scale's dimensions ranged from acceptable to good, as indicated by Cronbach's α. The section on "Technological literacy and knowledge of informatics and AI" registered an α of 0.718, while the section "Current state of AI in the curriculum and preferences for AI education" scored an α of 0.726, both displaying acceptable internal consistency. A Cronbach's α value of 0.825 for the "Perspectives towards AI in the medical profession" section denoted good internal consistency. The Kaiser–Meyer–Olkin measure for sampling adequacy was 0.801, confirming the sample's representational validity. Bartlett's test of sphericity returned a *P*-value of less than 0.001, validating the chosen method for factor analysis. Factor analysis yielded a structure comprising 15 items across three dimensions, collectively explaining 54% of the total variance. Factor loadings for individual items ranged from 0.495 for "Which of these technical devices do you use at least once a week?" to 0.888 for "What is your general attitude toward the application of artificial intelligence (AI) in medicine?".

### Study cohort

**Fig. 1:**
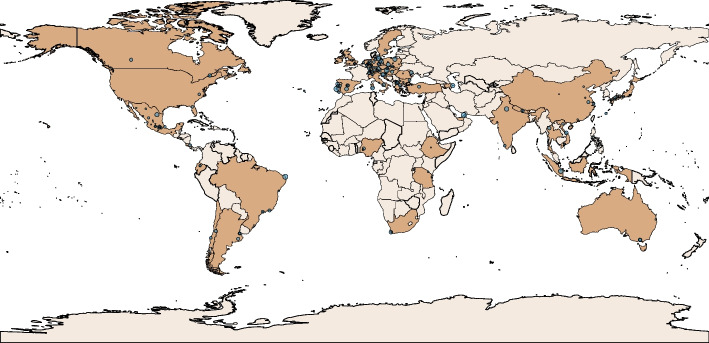
The world map displays the geographical distribution of participating institutions (blue dots) in relation to the number of respondents per institution

**Fig. 2:**
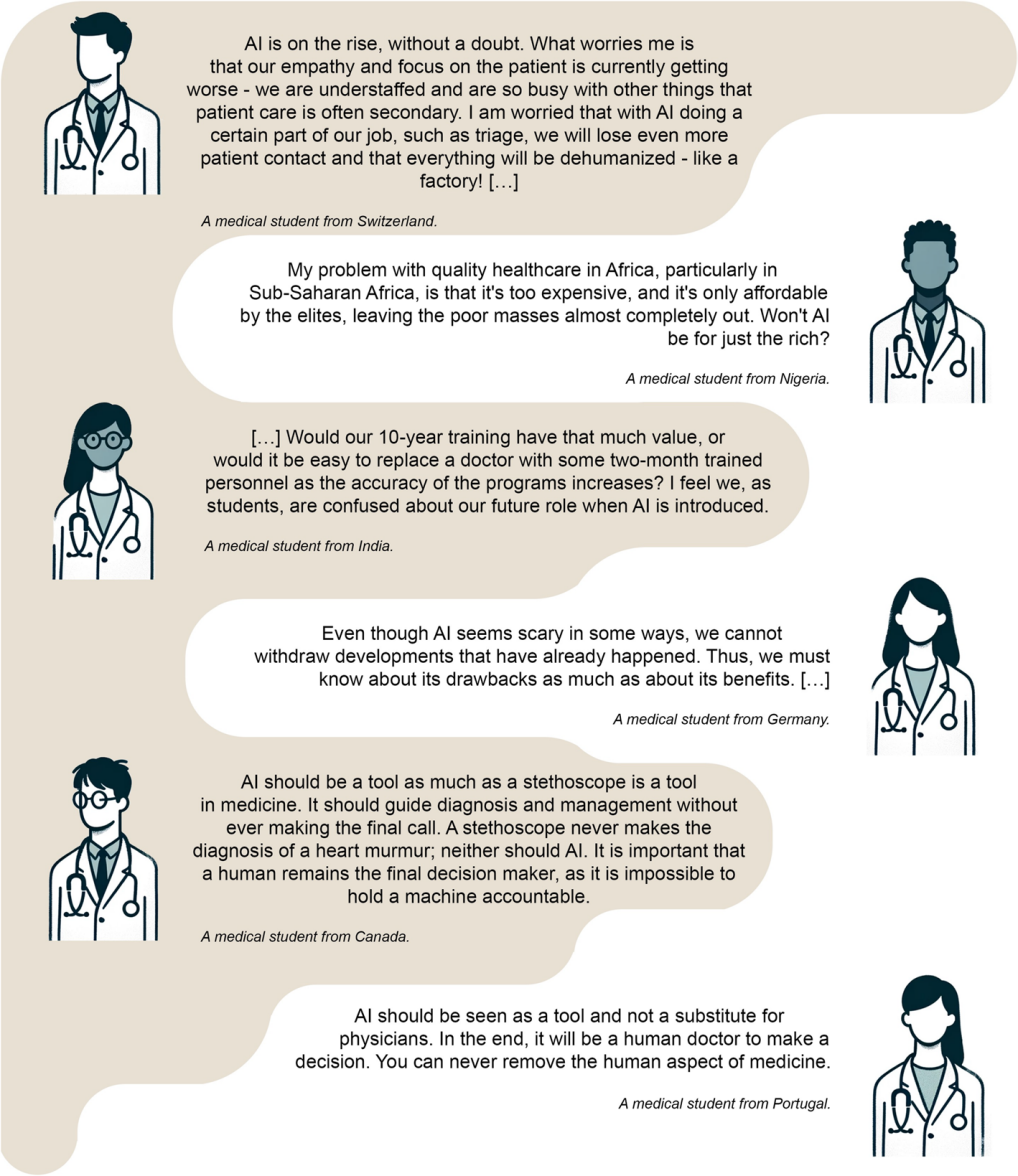
Diverse perspectives from medical students on the integration of artificial intelligence (AI) in healthcare education and practice. The selected quotes reflect a range of sentiments, from concerns about dehumanization and potential challenges in low-resource settings to viewing AI as a beneficial tool that complements rather than replaces the human touch in medicine

Between the first of April and the first of October 2023, 4900 responses were recorded, of which 4345 (88.7%) were collected via the English survey and 555 (11.3%) via the Spanish survey version. Of these, 283 (5.8%) respondents reported degrees other than medicine, dentistry, or veterinary medicine or indicated that they had completed their studies, while 21 (0.4%) did not respond to any multiple-choice item or did not indicate their degree. The final study cohort comprised 4596 participants from 192 faculty and 48 countries, of whom 4313 (93.8%) were medical, 205 (4.5%) dentistry, and 78 (1.7%) veterinary medicine students. Of 5,575,307 enrolled students from all degrees at the 183 (95.3%) participating faculties in which the total enrollment number was publicly available, the survey achieved an average response rate of 0.2% (standard deviation: 0.4%). Most respondents studied in Southern/Eastern European (*n* = 1240, 27%) countries, followed by Northern/Western Europe (*n* = 1110, 24.2%), Asia (*n* = 944, 20.5%), South America (*n* = 555, 12.1%), North America (*n* = 515, 11.2%), Africa (*n* = 125, 2.7%), and Australia (*n* = 104, 2.3%). Please refer to Fig. 1 to view the distribution of participating institutions in relation to the number of participants on a world map. A detailed list of survey participants divided by country, faculty, city, degree, number of enrolled students, and response rate is provided in the appendix (see Supplementary Table 8). The median age of the study population was 22 years (IQR: 20–24 years). 56.6% of the participants were female (*n* = 2600) and 42.4% male (*n* = 1946), with a median academic year of 3 (IQR: 2–5 years). Full descriptive data, including items on technological literacy and preferences for AI teaching in the medical curriculum, are displayed in Table 2. Any free field comments of the survey participants are listed in the appendix (see Supplementary Table 9), with selected comments highlighted in Fig. 2.

**Table 2:** Descriptive data of the study population and results of the questions about tech-savviness and topic preferences for AI teaching

Item	Value (*N* = 4596)
**Gender**	
Respondents, n (% of N)	4594 (99.96)
Female, n (% of respondents)	2600 (56.6)
Male, n (% of respondents)	1946 (42.36)
Diverse, n (% of respondents)	25 (0.54)
Prefer not to disclose, n (% of respondents)	23 (0.5)
**Age**	
Respondents, n (% of N)	4571 (99.46)
Years, median (IQR)	22 (20–24)
**Current academic year**	
Respondents, n (% of N)	4473 (97.32)
Years, median (IQR)	3 (2–5)
**Total academic years**	
Respondents, n (% of N)	4315 (93.89)
Years, median (IQR)	6 (6–6)
**Which of these technical devices do you use at least once a week?**	
Respondents, n (% of N)	4596 (100)
Smartphone, n (% of respondents)	4406 (95.87)
PC/laptop, n (% of respondents)	4020 (87.47)
Game console (e.g., PlayStation, Switch), n (% of respondents)	511 (11.12)
Tablet (e.g., iPad), n (% of respondents)	2172 (47.26)
E-reader, n (% of respondents)	325 (7.07)
Smartwatch, n (% of respondents)	1033 (22.48)
None, n (% of respondents)	9 (0.2)
**Have you already programmed code?**^**a**^	
Respondents, n (% of N)	4585 (99.76%)
Yes, n (% of respondents)	912 (19.89)
C, n (% of respondents)	85 (1.85)
C + + , n (% of respondents)	155 (3.38)
C#, n (% of respondents)	37 (0.81)
CSS, n (% of respondents)	32 (0.7)
HTML, n (% of respondents)	107 (2.33)
Java, n (% of respondents)	166 (3.62)
JavaScript, n (% of respondents)	91 (1.98)
MATLAB, n (% of respondents)	35 (0.76)
Pascal, n (% of respondents)	17 (0.37)
PHP, n (% of respondents)	24 (0.52)
Python, n (% of respondents)	382 (8.33)
R, n (% of respondents)	284 (6.19)
SQL, n (% of respondents)	11 (0.24)
Visual Basic, n (% of respondents)	14 (0.31)
No, n (% of respondents)	3673 (80.11)
**What would you like to learn about artificial intelligence (AI) as part of your medical curriculum?**	
Respondents, n (% of N)	4596 (100)
Theory and background (e.g., mathematical basics), n (% of respondents)	1549 (33.7)
Practical skills (e.g., learning programming languages; solving medical problems with AI), n (% of respondents)	3515 (76.48)
History and development, n (% of respondents)	827 (17.99)
Legal and ethical aspects, n (% of respondents)	2518 (54.79)
Future perspectives of AI in medicine, n (% of respondents)	3278 (71.32)
No preference, n (% of respondents)	162 (3.52)
None, n (% of respondents)	12 (0.26)

^a^To enhance data presentation, programming languages with a sample size of fewer than 10 respondents were omitted

Abbreviation: *IQR*, Interquartile range

### Collective perceptions towards artificial intelligence

**Fig. 3:**
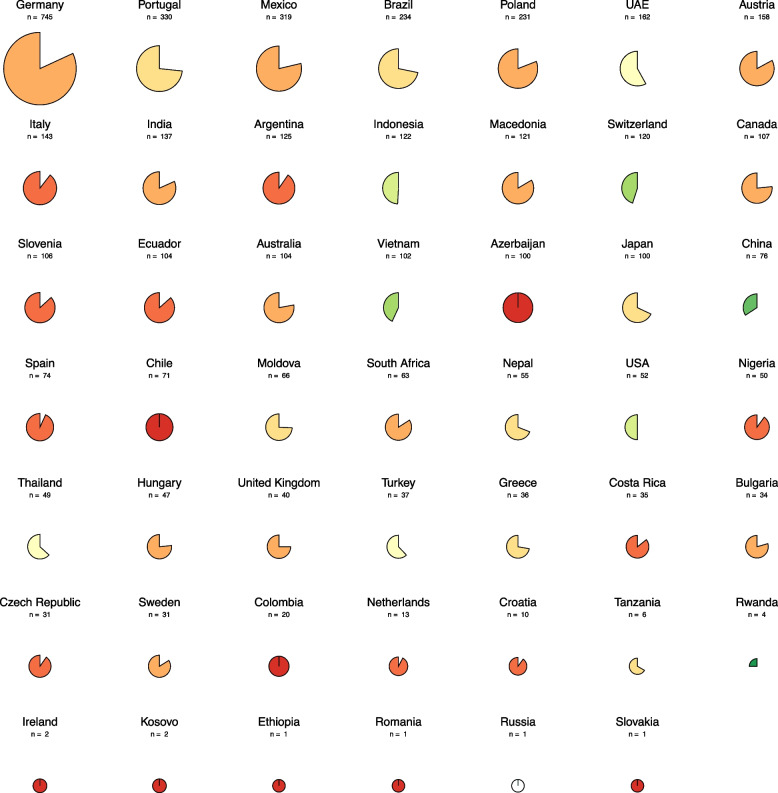
Pie charts illustrating student responses at the country level for the item "As part of my studies, there are curricular events on artificial intelligence (AI) in medicine.". A more filled, darker red chart indicates a higher proportion of students reporting no AI events, while a less filled, greener chart indicates fewer students reporting the absence of AI events. The missing portion of each chart displays the proportion of students who reported AI events, regardless of the duration. An all-white pie chart indicates that all students reported AI events in the medical curriculum. The absolute number of responses per country is shown above each chart. Analysis of the pie charts from countries with a representative sample of at least 50 respondents reveals that, among 28 nations, only four (Indonesia, Switzerland, Vietnam, and China) exhibited over 50% of students reporting the inclusion of AI events within their medical curriculum. Data from the USA displayed an equal proportion of students reporting the presence or absence of AI events in their curriculum (50% each). The residual 23 countries, encompassing Germany, Portugal, Mexico, Brazil, Poland, UAE, Austria, Italy, India, Argentina, Macedonia, Canada, Slovenia, Ecuador, Australia, Azerbaijan, Japan, Spain, Chile, Moldova, South Africa, Nepal, and Nigeria, had a lower proportion of students reporting the integration of AI in the medical curriculum. Abbreviations: UAE, United Arab Emirates; USA, United States of America

**Fig. 4:**
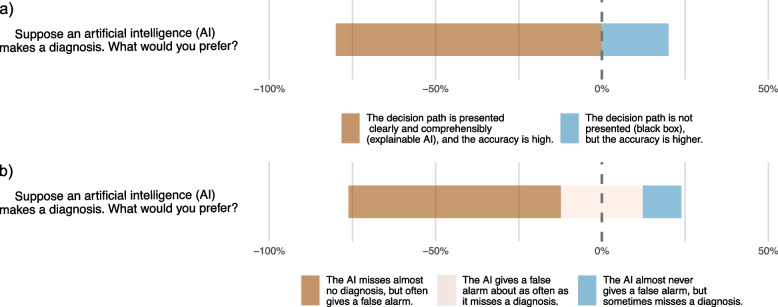
Gantt diagrams depicting medical students' preferences in AI diagnostics. **a** AI explainability (*n* = 3659, 80.2%) versus higher accuracy (*n* = 902, 19.8%) and **b**) higher sensitivity (*n* = 2906, 63.9%) versus higher specificity (*n* = 1118, 24.6%) or equal sensitivity and specificity (*n* = 524, 11.5%)

Table 3 displays the survey results for Likert scale items. Students generally reported a rather or extremely positive attitude towards the application of AI in medicine (3091, 67.6%). The highest positive attitude towards AI in the medical profession was recorded for the item "How do you estimate the effect of artificial intelligence (AI) on the efficiency of healthcare processes in the next 10 years?" with 4042 respondents (88.4%) estimating a moderate or great improvement. Contrarily, 3171 students (69.4%) rather or completely agreed with the item "The use of artificial intelligence (AI) in medicine will increasingly lead to legal and ethical conflicts.". Regarding AI education and knowledge, 3451 students (75.3%) reported no or little knowledge of AI, and 3474 (76.1%) rather or completely agreed that they would like to have more teaching on AI in medicine as part of their curricula. On the other hand, 3497 (76.3%) students responded that they did not have any curricular events on AI as part of their degree, as illustrated on the country level in Fig. 3. Variability in responses was observed, ranging from 0.279 for the item "How would you rate your general knowledge of artificial intelligence (AI)?" —measured on a four-point Likert scale— to 1.372 for "With my current knowledge, I feel sufficiently prepared to work with artificial intelligence (AI) in my future profession as a physician.". Notably, the items capturing the trade-offs in medical AI diagnostics revealed that most students preferred AI explainability (*n* = 3659, 80.2%) over a higher accuracy (*n* = 902, 19.8%) and higher sensitivity (*n* = 2906, 63.9%) over higher specificity (*n* = 1118, 24.6%) or equal sensitivity/specificity (*n* = 524, 11.5%), as visualized in Fig. 4.

**Table 3:** Survey results of Likert scale format items on attitudes towards the medical degree, AI in the medical profession, AI education and knowledge

Item	n (*N* = 4596)	Variance
**What is your current general attitude toward your medical studies?**		
Respondents (% of N)	4579 (99.63)	
Extremely negative (% of respondents)	48 (1.05)	0.638
Rather negative (% of respondents)	216 (4.72)	
Neutral (% of respondents)	899 (19.63)	
Rather positive (% of respondents)	2597 (56.72)	
Extremely positive (% of respondents)	819 (17.89)	
**What is your general attitude toward the application of artificial intelligence (AI) in medicine?**		
Respondents (% of N)	4576 (99.56)	
Extremely negative (% of respondents)	60 (1.31)	0.748
Rather negative (% of respondents)	307 (6.71)	
Neutral (% of respondents)	1118 (24.43)	
Rather positive (% of respondents)	2286 (49.96)	
Extremely positive (% of respondents)	805 (17.59)	
**How do you estimate the effect of artificial intelligence (AI) on the efficiency of healthcare processes in the next 10 years?**		
Respondents (% of N)	4570 (99.43)	
Great deterioration (% of respondents)	48 (1.05)	0.610
Moderate deterioration (% of respondents)	194 (4.25)	
No effect (% of respondents)	286 (6.26)	
Moderate improvement (% of respondents)	2687 (58.8)	
Great improvement (% of respondents)	1355 (29.65)	
**The use of artificial intelligence (AI) in medicine will increasingly lead to legal and ethical conflicts**		
Respondents (% of N)	4575 (99.54)	
Completely disagree (% of respondents)	73 (1.6)	0.944
Rather disagree (% of respondents)	370 (8.09)	
Neutral (% of respondents)	961 (21.01)	
Rather agree (% of respondents)	1869 (40.85)	
Completely agree (% of respondents)	1302 (28.46)	
**What is your view on the influence of artificial intelligence (AI) on the profession of physicians? AI will affect the everyday life of physicians in a way that is…**		
Respondents (% of N)	4571 (99.56)	
Extremely negative (% of respondents)	50 (1.09)	0.630
Rather negative (% of respondents)	323 (7.07)	
Neutral (% of respondents)	1027 (22.47)	
Rather positive (% of respondents)	2676 (58.54)	
Extremely positive (% of respondents)	495 (10.83)	
**How would you rate artificial intelligence (AI) software being available to physicians as a second opinion on medical issues?**		
Respondents (% of N)	4565 (99.33)	
Extremely negative (% of respondents)	89 (1.95)	0.842
Rather negative (% of respondents)	428 (9.38)	
Neutral (% of respondents)	1026 (22.48)	
Rather positive (% of respondents)	2281 (49.97)	
Extremely positive (% of respondents)	741 (16.23)	
**I think working with artificial intelligence (AI) as a physician is necessary to stay competitive**		
Respondents (% of N)	4576 (99.56)	
Completely disagree (% of respondents)	135 (2.95)	0.998
Rather disagree (% of respondents)	523 (11.43)	
Neutral (% of respondents)	1115 (24.37)	
Rather agree (% of respondents)	1988 (43.44)	
Completely agree (% of respondents)	815 (17.81)	
**With my current knowledge, I feel sufficiently prepared to work with artificial intelligence (AI) in my future profession as a physician**		
Respondents (% of N)	4577 (99.59)	
Completely disagree (% of respondents)	1003 (21.91)	1.372
Rather disagree (% of respondents)	1649 (36.03)	
Neutral (% of respondents)	910 (19.88)	
Rather agree (% of respondents)	740 (16.17)	
Completely agree (% of respondents)	275 (6.01)	
**I would like to have more teaching on artificial intelligence (AI) in medicine as part of my studies**		
Respondents (% of N)	4565 (99.33)	
Completely disagree (% of respondents)	86 (1.88)	0.831
Rather disagree (% of respondents)	191 (4.18)	
Neutral (% of respondents)	814 (17.83)	
Rather agree (% of respondents)	2033 (44.53)	
Completely agree (% of respondents)	1441 (31.57)	
**As part of my studies, there are curricular events on artificial intelligence (AI) in medicine**		
Respondents (% of N)	4581 (99.67)	
No (% of respondents)	3497 (76.34)	0.458
Yes; 1–5 h in total (% of respondents)	820 (17.9)	
Yes; > 5–10 h in total (% of respondents)	178 (3.89)	
Yes; > 10–20 h in total (% of respondents)	48 (1.05)	
Yes; > 20 h in total (% of respondents)	38 (0.83)	
**How would you rate your general knowledge of artificial intelligence (AI)?**		
Respondents (% of N)	4585 (99.76)	
No knowledge (never heard of AI). (% of respondents)	170 (3.71)	0.279
Little knowledge (e.g., documentary seen on TV). (% of respondents)	3281 (71.56)	
Good knowledge (e.g., read several journal articles on AI). (% of respondents)	1064 (23.21)	
Expert (e.g., involved in AI research/development). (% of respondents)	70 (1.53)

### Regional comparisons

Please refer to Table 4 to view the results of the comparison of responses from the Global North and South for Likert scale format items. Perceptions between the Global North and South differed significantly for nine Likert scale format items. The highest effect size was observed for the item on AI increasing ethical and legal conflicts, with respondents from the Global North indicating a higher agreement (median: 4, IQR: 3–5) compared to those from the Global South (median: 4, IQR: 3–4; *r* = 0.185; *P* < 0.001). Notably, Global South students felt more prepared to use AI in their future practice (median: 3, IQR: 2–4) compared to their Global North counterparts (median: 2, IQR: 1–3; *r* = 0.162; *P* < 0.001) and reported longer AI-related curricular events (median: 1, IQR: 1–2; Global North: median: 1, IQR: 1–1; *r* = 0.090; *P* < 0.001). Conversely, Global North students rated their AI knowledge higher (median: 2, IQR: 2–3; Global South: median: 2, IQR: 2–2; *r* = 0.025; *P* < 0.001).

**Table 4:** Regional comparison of respondents from the Global North and South for Likert scale format items

Item	n (% of *N* = 4596)	Median (IQR)	*P*^*a*^	*r*
**Q1**				
Global North	3162 (68.8)	4 (4–4)	< .001	.095
Global South	1417 (30.83)	4 (3–4)		
**Q2**				
Global North	3164 (68.84)	4 (3–4)	.71	.006
Global South	1412 (30.72)	4 (3–4)		
**Q3**				
Global North	3155 (68.85)	4 (4–5)	< .001	.061
Global South	1415 (30.79)	4 (4–5)		
**Q4**				
Global North	3159 (68.73)	4 (3–5)	< .001	.185
Global South	1416 (30.81)	4 (3–4)		
**Q5**				
Global North	3157 (68.69)	4 (3–4)	.002	.046
Global South	1414 (30.77)	4 (3–4)		
**Q6**				
Global North	3155 (68.65)	4 (3–4)	.02	.035
Global South	1410 (30.68)	4 (3–4)		
**Q7**				
Global North	3160 (68.76)	4 (3–4)	< .001	.049
Global South	1416 (30.81)	4 (3–4)		
**Q8**				
Global North	3161 (68.78)	2 (1–3)	< .001	.162
Global South	1416 (30.81)	3 (2–4)		
**Q9**				
Global North	3155 (68.65)	4 (4–5)	.11	.024
Global South	1410 (30.68)	4 (3–5)		
**Q10**				
Global North	3165 (68.86)	1 (1–1)	< .001	.090
Global South	1416 (30.81)	1 (1–2)		
**Q11**				
Global North	3167 (68.91)	2 (2–3)	< .001	.025
Global South	1418 (30.85)	2 (2–2)		

^a^Compares student responses in the Global North and South using the Mann–Whitney U-test

*Abbreviations IQR* Interquartile range, Q1, What is your current general attitude toward your medical studies?; Q2, What is your general attitude toward the application of artificial intelligence (AI) in medicine?; Q3, How do you estimate the effect of artificial intelligence (AI) on the efficiency of healthcare processes in the next 10 years?; Q4, The use of artificial intelligence (AI) in medicine will increasingly lead to legal and ethical conflicts.; Q5, What is your view on the influence of artificial intelligence (AI) on the profession of physicians? AI will affect the everyday life of physicians in a way that is…; Q6, How would you rate artificial intelligence (AI) software being available to physicians as a second opinion on medical issues?; Q7, I think working with artificial intelligence (AI) as a physician is necessary to stay competitive.; Q8, With my current knowledge, I feel sufficiently prepared to work with artificial intelligence (AI) in my future profession as a physician.; Q9, I would like to have more teaching on artificial intelligence (AI) in medicine as part of my studies.; Q10, As part of my studies, there are curricular events on artificial intelligence (AI) in medicine.; Q11, How would you rate your general knowledge of artificial intelligence (AI)?

For continental comparison, the Kruskal–Wallis one-way analysis of variance revealed significantly different Likert scale responses across all survey items (see Table 5). Subsequent Dunn-Bonferroni post hoc analysis displayed various significant differences in Likert scale responses for pairwise regional comparisons, while median and IQR remained largely consistent. Considering only medium to large effect sizes, the item "The use of artificial intelligence (AI) in medicine will increasingly lead to legal and ethical conflicts." yielded an *r* of 0.301 when comparing Northern/Western European (median: 4, IQR: 4–5) and South American participants (median: 4, IQR: 3–4; *P* < 0.001), and an *r* of 0.311 between South American and Australian participants (median: 4, IQR: 4–5; *P* < 0.001). Similarly, the statement "With my current knowledge, I feel sufficiently prepared to work with artificial intelligence (AI) in my future profession as a physician." displayed strong effect sizes in comparisons between North/West Europe (median: 2, IQR: 1–2) and Asia (median: 3, IQR: 2–4; *r* = 0.531; *P* < 0.001), South/East Europe (median: 2, IQR: 2–3) and Asia (*r* = 0.342; *P* < 0.001), and South America (median: 2, IQR: 2–3) and Asia (*r* = 0.398; *P* < 0.001).

**Table 5:** Regional comparison of Likert scale format items on the continental level

Item	n (% of *N* = 4596)	Median (IQR)	*P*^*a*^
**Q1**			
North/Western Europe	1108 (24.11)	4 (4–4)	< .001^b^
South/Eastern Europe	1230 (26.76)	4 (4–4)	
Asia	941 (20.47)	4 (3–4)	
North America	514 (11.18)	4 (4–4)	
South America	555 (12.08)	4 (3–4)	
Africa	124 (2.7)	4 (4–5)	
Australia	104 (2.26)	4 (4–4)	
**Q2**			
North/Western Europe	1109 (24.13)	4 (3–4)	< .001^c^
South/Eastern Europe	1231 (26.78)	4 (3–4)	
Asia	937 (20.39)	4 (3–4)	
North America	514 (11.18)	4 (3–4)	
South America	554 (12.05)	4 (4–4)	
Africa	124 (2.7)	4 (3–4)	
Australia	104 (2.26)	4 (3–4)	
**Q3**			
North/Western Europe	1107 (24.09)	4 (4–5)	.001^d^
South/Eastern Europe	1223 (26.61)	4 (4–5)	
Asia	939 (20.43)	4 (4–5)	
North America	515 (11.21)	4 (4–5)	
South America	555 (12.08)	4 (4–5)	
Africa	124 (2.7)	4 (4–5)	
Australia	104 (2.26)	4 (4–4)	
**Q4**			
North/Western Europe	1108 (24.11)	4 (4–5)	< .001^e^
South/Eastern Europe	1227 (26.7)	4 (4–5)	
Asia	941 (20.47)	4 (3–4)	
North America	515 (11.21)	4 (3–4)	
South America	554 (12.05)	4 (3–4)	
Africa	124 (2.7)	4 (3–5)	
Australia	103 (2.24)	4 (4–5)	
**Q5**			
North/Western Europe	1109 (24.13)	4 (3–4)	< .001^f^
South/Eastern Europe	1225 (26.65)	4 (3–4)	
Asia	940 (20.45)	4 (3–4)	
North America	515 (11.21)	4 (3–4)	
South America	553 (12.03)	4 (4–4)	
Africa	124 (2.7)	4 (3–4)	
Australia	104 (2.26)	4 (3–4)	
**Q6**			
North/Western Europe	1108 (24.11)	4 (3–4)	< .001^ g^
South/Eastern Europe	1226 (26.68)	4 (3–4)	
Asia	936 (20.37)	4 (3–4)	
North America	513 (11.16)	4 (3–4)	
South America	553 (12.03)	4 (4–4)	
Africa	124 (2.7)	4 (3–4)	
Australia	103 (2.24)	4 (3–4)	
**Q7**			
North/Western Europe	1108 (24.11)	4 (3–4)	< .001^ h^

## Discussion

Our multicenter study of 4596 medical, dental, and veterinary students from 192 faculties in 48 countries provides crucial insights into the global landscape of AI perception and education in healthcare curricula. The findings reveal a nuanced picture: while students generally express optimism about AI’s role in future healthcare practice, this is tempered by significant concerns and a striking lack of preparedness.

The educational basis of our study lies in addressing a critical gap in AI education within medical curricula, exploring how this deficiency varies across different regions, particularly between continents and the Global North and South. As AI rapidly advances and promises to reshape healthcare, the need for future physicians to be adequately prepared through comprehensive AI education becomes increasingly urgent. Our study goes beyond merely asserting the necessity of AI education by elucidating regional differences in perceptions and experiences related to AI among healthcare students.

Our findings extend previous research highlighting inadequacies in AI education in medical schools globally. Kolachalama and Garg [36] noted that AI is not widely taught in medical schools, with most curricula lacking substantial AI training modules. Chan and Zary [37] reinforced this, emphasizing the gap between recognizing AI’s potential benefits and actually integrating AI education into medical programs. Our study confirms these deficiencies on a larger, international scale, revealing that over three-quarters of students reported no AI-related events in their curriculum, despite strong interest in such education. Importantly, our research uncovers regional disparities in AI education and perception.

Students from the Global South were generally less likely to report having AI incorporated into their curricula compared to their counterparts in the Global North. This discrepancy underscores the need for tailored educational strategies that consider these regional differences to ensure equitable preparation for an AI-enhanced medical landscape. The observed differences in perceived preparedness for working with AI, particularly among Asian students, may reflect varying national AI policies, educational strategies, and macroeconomic factors [38, 39].

Depending on the study and item design, self-reported AI knowledge in the literature ranges from 2.8% of 2981 medical students in Turkey in 2022 who reported feeling informed about the use of AI in medicine to 51.8% of 900 medical students in Jordan in 2021 who indicated having read articles about AI or machine learning in the past two years [21, 40–44]. On the other hand, the reported prevalence of AI training in the medical curriculum ranges, for instance, from 9.2% in a 2020 survey of 484 medical students in the United Kingdom up to 24.4% in a 2022 study among 2981 medical students in Turkey, although variations in item designs and demographic contexts hinder a comprehensive longitudinal analysis [22, 40, 42, 43, 45]. In our study, less than 18% (*n* = 5) of countries with a sample size of 50 or more participants had a higher or equal proportion of students reporting any duration of AI teaching, pointing to a persistent deficit in medical AI education across various demographic landscapes. Overall, the incorporation of AI into medical education on a broader national or international scale is limited, and the adoption of frameworks, certification programs, interdisciplinary collaborations, modules, and formal lectures seems still to be at an early stage [14, 46–48, 49].

While our study design and varying sample sizes across regions complicate causal analysis, the fact that three of four countries with over 50% of students reporting AI training were in Asia suggests a potential link between educational exposure and perceived readiness.

Despite the overall positive outlook, our study reveals a pronounced concern among students about the ethical and legal challenges posed by AI integration in healthcare. This echoes findings from Mehta et al. and Civaner et al. [40, 50], highlighting the critical need for AI education to address not only technical skills but also ethical, legal, and societal implications.

In terms of educational preferences, most of the participants in our study indicated their interest in learning practical skills, followed by future perspectives and legal and ethical aspects of medical AI. This underscores the great potential of AI education to not only improve medical students' oversight, knowledge, and practical skills in using AI but also to educate about ethical, legal, and societal implications — topics that are also addressed in other AI education frameworks, such as the United Nations Educational, Scientific and Cultural Organization K-12 AI curricula report [51].

In our subgroup analysis of respondents across continents, two items displayed moderate to large effect sizes. First, participants from South America were less likely to agree that the use of medical AI will increase ethical and legal conflicts compared to participants from Northern/Western Europe and Australia. Yet, students' median responses in these regions were identical. Thus, the level of effect size primarily reflects outliers rather than a uniform regional disparity in opinion. Second, Asian students reported being better prepared to work with AI in their future careers. Although these differences in perceived preparedness could be driven by different national AI policies and educational strategies as well as macroeconomic factors, our study design and varying sample sizes across regions complicate a causal analysis [38, 39].

Finally, the strong preference for explainable AI systems over highly accurate but opaque ones underscores the growing emphasis on ‘Explainable AI’ in medicine, underlining the importance of transparency in fostering trust and acceptance among future healthcare professionals [52–54, 55].

This study has limitations. First, the uneven regional distribution of participants potentially biased results in favor of overrepresented regions. In addition, the online design and language availability in either English or Spanish, as well as the non-probability convenience sampling method, may have introduced selection bias by excluding students without internet access, students who were not proficient in either language, or students who did not wish to participate. Another potential source of selection bias could be that respondents with a specific interest in or experience with AI were more likely to participate in the survey. Furthermore, the calculated response rate appeared to be rather low due to the lack of data on the number of students enrolled in each medical discipline for most participating institutions. Consequently, we derived the response rate using the total student enrollment numbers, which significantly underestimated the true rate of participation among medical students as it assumes that all students within each faculty received an invitation to participate. Moreover, the presence of 20 institutions with fewer than 50 student respondents has skewed the response rate further downward.

## Conclusions

In conclusion, our study -the currently largest survey of medical students’ perceptions towards AI in healthcare education and practice- reveals a broadly optimistic view of AI’s role in healthcare. It draws on insights from students with diverse geographical, sociodemographic, and cultural backgrounds, underlining the critical need for AI education in medical curricula around the world and identifying a universal challenge and opportunity: to adeptly prepare healthcare students for a future that integrates AI into healthcare practice.

## Supplementary Information

Supplementary Material 1.

Supplementary Material 2.

## Data Availability

All data collected and analyzed as part of this study is available at figshare at: 10.6084/m9.figshare.24422422.

## Abbreviations

AI: Artificial intelligence
AMEE: Association for Medical Education in Europe
ChatGPT: Chat Generative Pre-trained Transformers
IQR: Interquartile range
LLM: Large language model
REDCap: Research Electronic Data Capture
STROBE: Strengthening the reporting of observational studies in epidemiology
USMLE: United States Medical Licensing Examination
