# Supplementary material for: Global cross-sectional student survey on AI in medical, dental, and veterinary education and practice at 192 faculties
Source: BMC Med Educ. 2024 Sep 28;24:1066. doi: 10.1186/s12909-024-06035-4 (PMC11439199; doi:10.1186/s12909-024-06035-4)
Supplement: Supplementary file 1 — Supplementary Material 1. [file 12909_2024_6035_MOESM1_ESM.pdf]

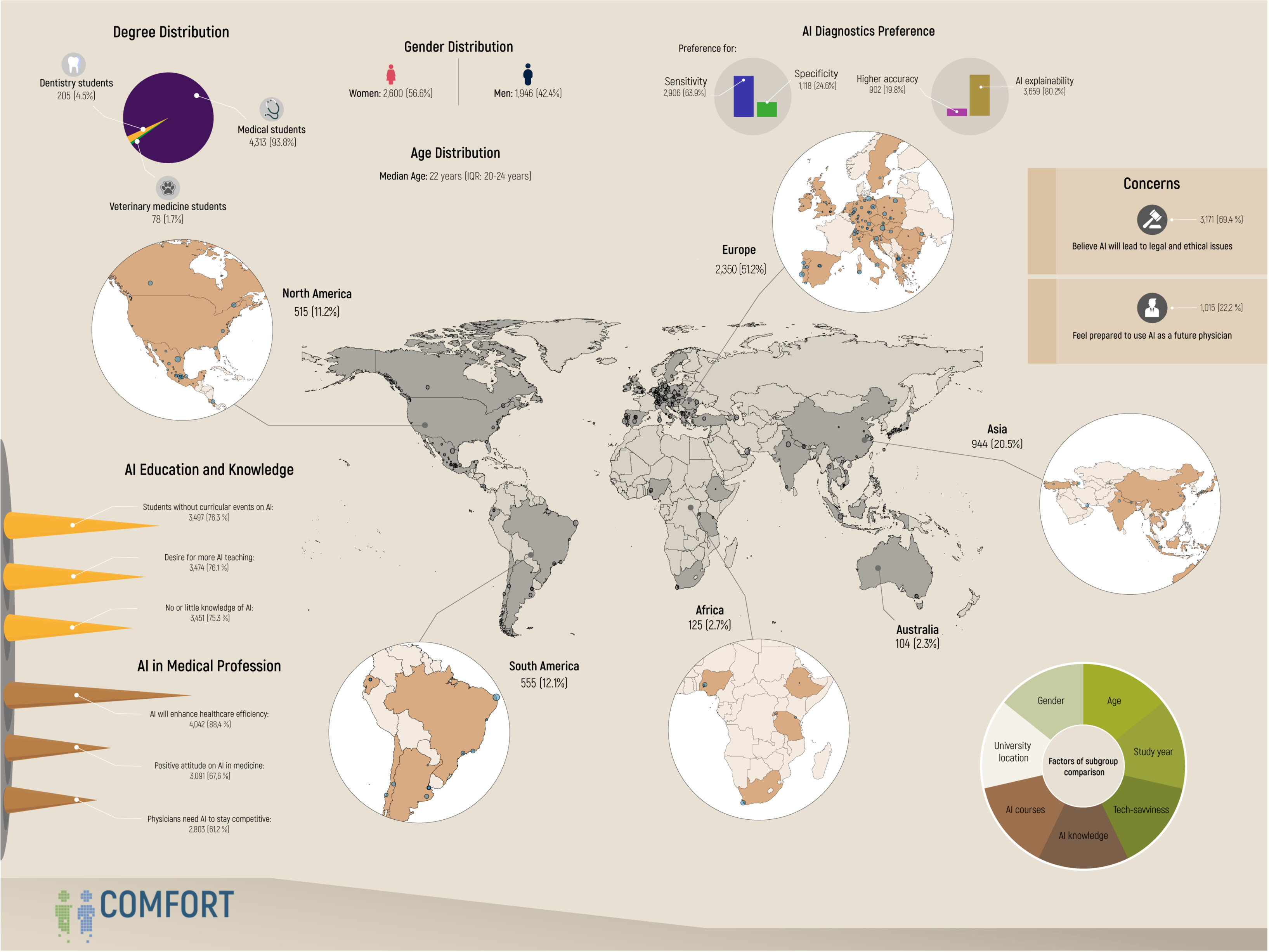

Degree Distribution
Dentistry students
205 (4.5%)
Medical students
4,313 (93.8%)
Veterinary medicine students
78 (1.7%)
Gender Distribution
Women: 2,600 (56.6%)
Men: 1,946 (42.4%)
Age Distribution
Median Age: 22 years (IQR: 20-24 years)
AI Diagnostics Preference
Preference for:
Sensitivity
2,906 (63.9%)
Specificity
1,118 (24.6%)
Higher accuracy
902 (19.8%)
AI explainability
3,659 (80.2%)
Concerns
3,171 (69.4 %)
Believe AI will lead to legal and ethical issues
1,015 (22.2 %)
Feel prepared to use AI as a future physician
North America
515 (11.2%)
Europe
2,350 (51.2%)
Asia
944 (20.5%)
Africa
125 (2.7%)
Australia
104 (2.3%)
South America
555 (12.1%)
AI Education and Knowledge
Students without curricular events on AI:
3,497 (76.3 %)
Desire for more AI teaching:
3,474 (76.1 %)
No or little knowledge of AI:
3,451 (75.3 %)
AI in Medical Profession
AI will enhance healthcare efficiency:
4,042 (88.4 %)
Positive attitude on AI in medicine:
3,091 (67.6 %)
Physicians need AI to stay competitive:
2,803 (61.2 %)
Factors of subgroup comparison
Gender
Age
Study year
Tech-savviness
AI knowledge
AI courses
University location
COMFORT
